# New Appliances and Things Medical

**Published:** 1894-08-25

**Authors:** 


### NEW APPLIANCES AND THINGS MEDICAL.

[All preparations, appliances, novelties, &c., of which a notice is desired, should be sent for the Editor, to care of The Manager, 428, Strand, London, W.C.]

### IZAL TOILET AND MEDICAL PREPARATIONS.

(Newton, Chambers, and Co., Limited, Thorncliffe, near Sheffield.)

The value of Izal having been fully recognised by the medical profession, Messrs. Newton, Chambers and Co., Ltd., wisely striking while the iron is hot, have extended its applicability for general use by the production of a number of toilet and medical preparations containing percentages of this powerful antiseptic. Of the samples sent us by the above firm, we have had an opportunity of making a practical trial of those which are most likely to find favour with the public and the profession, and of these we believe that the Izal toilet soap is destined to hold the foremost place. This soap is of the super-fatted variety, readily forms a smooth and silky lather, and, although powerfully antiseptic, has an odour which is distinctly agreeable to the user. There can be no doubt that, whether used merely for the face and hands or whether applied to a more extensive surface of the body in the bath, it must have a most destructive effect on the bacteria of the skin, thus to some extent preventing acne spots or pimples, and in certain cases preventing the more serious consequences of infection from pathogenic germs, and from the fomites of the eruptive exanthemata, from whose ravages we can never render ourselves safe. Izal tooth powder consists of a vehicle such as is generally used in similar powders, strongly impregnated with Izal, rendering the preparation at once a cleanser and an antiseptic for the teeth and gums, the two essentials for preserving the teeth from decay. Izal tooth powder we confidently recommend as a first-class preparation. Izal cream has also much to recommend it. It is a carefully prepared and agreeable emollient, peculiarly suitable for lubricating the skin after an attack of scarlatina or measles during the period of disquamation, and being pleasantly scented and non-poisonous, it answers the purpose of an excellent lip salve or application to chapped hands or superficial sores or abrasions. Izal lozenges render the mouth fauces and pharynx antiseptic, and consequently will do much to protect a person from infection, should he in any way expose himself to sources of contamination; for, as is well known, it is through the mucous surfaces that most pathogenic germs effect an entrance into the system; and it is with the object of preventing infection through the nasal mucous membrane the Izal smelling salts have been prepared. The substitution of a smelling salt of the antiseptic class is a wise precaution for all those who habitually use this form of stimulant, since the usual object of smelling salts is in this case combined with a powerful antiseptic action.

